# Synthesis and hepatoprotective properties of *Acanthus ilicifolius* alkaloid A and its derivatives

**DOI:** 10.3892/etm.2013.1189

**Published:** 2013-06-28

**Authors:** LIN LIU, HUI FAN, PING QI, YAN MEI, LIJUAN ZHOU, LIPING CAI, XING LIN, JUN LIN

**Affiliations:** 1 Guangxi Medical University, Nanning, Guangxi 530021, P.R. China; 2 Shanghai Jiaotong University, Shanghai 200240, P.R. China; 3 The First Affiliated Hospital of Zhejiang University, Hangzhou, Zhejiang 310003, P.R. China

**Keywords:** *Acauthus ilicifolius* alkaloid A, synthesis, hepatoprotective potential, acute liver injury

## Abstract

*Acanthus ilicifolius* alkaloid A (4-hydroxy-2(3H)benzoxazolone, HBOA) is a naturally occurring compound that has been separated from *Acanthus ilicifolius*. Previous studies have reported the beneficial effects of HBOA on HSC-T6 cells. This study was undertaken in order to synthesize HBOA and two of its derivatives, specifically, 4-acetoxy-2(3H)-benzoxazolone (AcO-BOA) and 3-acetyl-4-acetoxy-2-benzoxazolone (TC-3), and to investigate the hepatoprotective potentials of these three compounds on CCl_4_-induced liver injury in mice. HBOA was prepared from 2-nitroresorcinol by a 'one pot' reduction and subsequent cyclization with urea. The acyl derivatives, AcO-BOA and TC-3, were prepared from HBOA using a substitution reaction. The compounds were synthesized with good yields (63.08–68.22%). An acute liver injury model was established by administering CCl_4_ intraperitoneally to Kunming mice. The mice were then intragastrically administered bifendate (150 mg/kg) or the synthesized compounds at three different doses (200, 100 and 50 mg/kg). The treatment with CCl_4_ was observed to increase the levels of aminotransferase (ALT), aspartate aminotransferase (AST), lactic dehydrogenase (LDH) and malondialdehyde (MDA) and decrease the levels of superoxide dismutase (SOD), catalase (CAT), glutathione (GSH) and glutathione peroxidase (Gpx) in the liver tissues of the mice. Furthermore, treatment with CCl_4_ elevated the expression level of the proinflammatory mediator TNF-α. However, HBOA and its derivatives attenuated the changes induced by CCl_4_. Furthermore, CCl_4_-induced histopathological changes were reduced by treatment with these compounds. These results suggest that HBOA and its acyl derivatives are able to significantly alleviate the hepatotoxicity induced by CCl_4_ in mice.

## Introduction

The liver is a vital organ in mammals, including rodents. Hepatotoxic agents may cause severe damage to hepatocytes; not only do they potentially catabolize radical-induced lipid peroxidation, damage the membranes of liver cells and organelles and cause swelling and necrosis of hepatocytes, but they may also induce an inflammatory response and release mediators of inflammation (1). CCl_4_ is used as a model agent for inducing acute liver injury in mice. CCl_4_-induced liver injury is associated with the aggravation of lipid peroxidation and the attenuation of antioxidant enzyme function (2). These changes result from the formation of reactive intermediates such as trichloromethyl (^•^CCl_3_) free radicals and reactive oxygen species (ROS) (3). Furthermore, CCl_4_-induced liver injury has been reported to cause significant increases in the levels of alanine aminotransferase (ALT), aspartate aminotransferase (AST), lactic dehydrogenase (LDH) and malondialdehyde (MDA) and reductions in the levels of superoxide dismutase (SOD), catalase (CAT), glutathione (GSH) and glutathione peroxidase (Gpx) (4). Therefore, reducing lipid peroxidation or oxidative stress may be an effective therapeutic strategy for preventing and treating CCl_4_-induced liver injury.

*Acanthus ilicifolius* L. (*Acanthaceae*) has spinous edges to its evergreen leaves and stipular spines at the stem nodes. It is a plant found in marshy habitats throughout the mangroves of China, including those of the Guangxi region and Guangdong and Fujian provinces, and in various Asian countries such as India, Burma and Thailand. In traditional Chinese medicine, *A. ilicifolius* is used to treat inflammation, hepatitis, swollen spleens, asthma, gastralgia and malignant tumors. Previous studies have reported that the crude alcoholic extract of *A. ilicifolius* exhibits antioxidant, hepatoprotective (5), antitumor (6) and antimicrobial activities (7).

*Acanthus ilicifolius* is protected in China. *Acanthus ilicifolius* alkaloid A (4-hydroxy-2(3H)-benzoxazolone, HBOA) has been separated from the *Acanthus ilicifolius* plant in the pharmaceutical chemistry laboratory of Guangxi Medical University. Moreover, in order to avoid the exploitation of the mangroves, we attempted to synthesize HBOA and successfully synthesized the core heterocyclic nucleus, 2(3H)-benzoxazolone (BOA) of HBOA. Benzoxazolone heterocycles are considered to be ‘privileged scaffolds’ in the design of pharmacological probes. Medicinal chemists have paid considerable attention to these units due to their capacity for mimicking phenol or catechol moieties in a metabolically stable template (8). Furthermore, 3-substituted BOA derivatives with more potent anti-inflammatory activity than aspirin have been reported (9). Therefore, HBOA, as a lead compound, may be modified to synthesize novel drugs with pharmacological activities.

**Figure 1:**
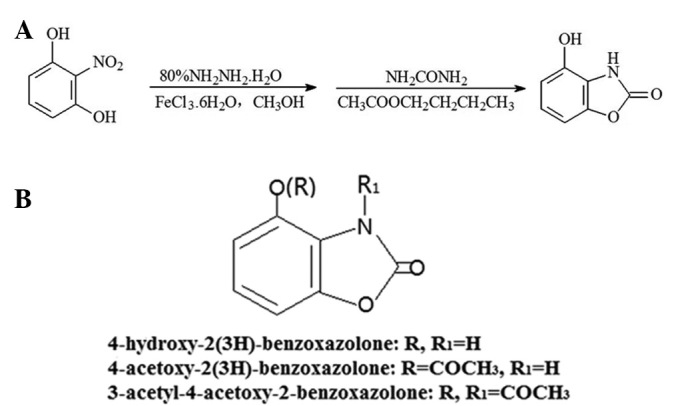
(A) Reaction scheme for 4-hydroxy-2(3H)-benzoxazolone (HBOA): 2-itroresorcinol is reduced and subsequently cyclized with urea in a ‘one-pot’ synthesis. (B) The chemical structure of HBOA and its derivatives. 4-Acetoxy-2(3H)-benzoxazolone (AcO-BOA) and 3-acetyl-4-acetoxy-2-benzoxazolone (TC-3) were prepared by HBOA acetylation.

There are a number of methods that may be used for the synthesis of BOA, including reactions between 2-aminophenol and urea phosgene or carbon monoxide; the reaction with urea is normally used in industry (10). A method of synthesizing HBOA from 2-aminoresorcinol hydrochloride and carbonyldiimidazole (CDI) by cyclization in dichloromethane, and a method for synthesizing 4-acetoxy-2(3H)-benzoxazolone (AcO-BOA) have been reported (11). The reaction scheme for HBOA is presented in Fig. 1A. Pharmacological studies of HBOA and its derivatives have been reported, and describe its use as an anti-inflammatory agent (12), analgesic (13,14), antioxidant, anti-convulsant and as a Type 2 diabetes agent (15–17). Our previous *in vitro* study demonstrated that HBOA and its derivatives possess hepatoprotective activity against the induction of apoptosis in hepatic stellate cells (HSC-T6), and the two acyl derivatives were shown to be more potent than HBOA (18). These findings prompted us to conduct the current study to investigate the hepatoprotective activity of HBOA and its acetylated derivatives in mice.

## Materials and methods

### Materials

Melting points (M.p.) were determined using an electrothermal melting point apparatus (Beijing Tech Instrument Co., Ltd., Beijing, China) and are uncorrected. The IR spectra of the compounds were recorded using a Spectrum One FT-IR spectrometer (Perkin-Elmer, Waltham, MA, USA). NMR (^1^H and ^13^C) spectra were recorded on a Bruker AV-500 MHz NMR spectrometer (Bruker BioSpin, Shanghai, China), and chemical shifts are expressed in parts per million (ppm, for δ) relative to tetramethylsilane (TMS) as an internal standard with CDCl_3_ as the solvent. Spin multiplets are provided as s (singlet), d (doublet), t (triplet), q (quartet) and m (multiplet). All compounds reported in the present study had IR, ^1^H and ^13^C-NMR data consistent with their structures. TLC analyses were performed on TLC plates (silica gel, GF_254_; Qingdao Haiyang Chemical Co., Ltd., Qingdao, China).

Bifendate (Bif) was purchased from Guangzhou Xingqun Pharmaceutical Co., Ltd. (Guangzhou, China); ALT, AST, LDH, MDA, SOD, CAT, GSH and Gpx testing kits were obtained from Nanjing Jiancheng Bioengineering Research Institute (Nanjing, China). The tumor necrosis factor-α (TNF-α) kit was obtained from Wuhan Boster Bio-engineering Co., Ltd (Wuhan, China).

### Methods

#### Synthesis of 2-nitroresorcinol

The intermediate product, 2-nitroresorcinol was synthesized by the reaction of resorcinol with a mixed acid consisting of concentrated sulfuric acid and nitric acid according to a method reported previously (19) (yield, 30.12%; M.p., 83–84°C).

#### Synthesis of HBOA

Activated carbon (18 g) was added to a solution of 8 g hexahydrated ferric chloride dissolved in 480 ml methanol and refluxed for ~30 min. To this mixture, 62 g 2-nitroresorcinol was added and the mixture was refluxed with stirring. Subsequently, 100 ml (75%) hydrazine hydrate was added dropwise to the mixture which was then refluxed for 4–5 h. The mixture was filtered to remove the activated carbon, and the solvent was evaporated under reduced pressure. Upon obtaining a homogenous paste, 620 ml ethyl acetate was added and the mixture was heated to 90°C. To the stirred mixture, 144 g urea was added and refluxing was conducted for 4–5 h. Following the removal of solvent under reduced pressure, 500 ml water was added and the mixture was refluxed twice for 30 min each. A crude product was obtained through filtration and subsequent drying. The product was extracted with n-butyl alcohol (3 × 800 ml). The extract was evaporated to dryness under reduced pressure and the resulting light brown crystals were filtered and recrystallized from ethanol (C_7_H_5_NO_3_; yield, 63.08%; M.p., 293-29°C). IR (KBr, cm^−1^), 3,289 (-OH, phenolic hydroxy), 1,741 (C=O, lactam) and 1,659 (C=O, amide); ^1^H-NMR (500 MHz; CDCl_3_) δ (ppm), 6.76 (1H, dd, H5), 6.74 (1H, dd, H7) and 6.94 (1H, m, H6).

#### Synthesis of AcO-BOA (TC-2)

To a stirred and cooled solution of 10.7 g HBOA in 750 ml acetone, at <5°C, was added 11.9 g triethylamine. Subsequently, a solution of 6.9 g acetyl chloride in 20 ml of acetone was added dropwise and the mixture was stirred for 5 h; the temperature did not rise above 5°C. Stirring proceeded for a further 5 h at room temperature and the mixture was left for a further 24 h. Following evaporation to dryness, the white precipitate of AcO-BOA was collected by filtration, washed with water, dried and recrystallized from water (C_9_H_7_NO_4_; yield, 68.22%; M.p., 191–193°C). IR (KBr, cm^−1^), 3,127 (-OH, phenolic hydroxy), 1,761 (C=O, lactam) and 1,639 (C=O, amide); ^1^H-NMR (500 MHz; CDCl_3_) δ (ppm), 7.15 (1H, dd, H5), 6.99 (1H, dd, H7), 7.17 (1H, m, H6) and 2.07 (3H, s, -CH_3_).

#### Synthesis of TC-3

To a solution of 11.0 g AcO-BOA in 750 ml acetone, which was stirred and cooled at <5°C, was added 11.9 g triethylamine. Then, 11.36 g acetyl chloride in 20 ml acetone was added dropwise. The reaction mixture was stirred for 2 h at <5°C. After stirring at room temperature for another day, the solvent was removed under reduced pressure and the residue was obtained in water and extracted with methenyl trichloride. Trichloromethane was recovered and white crystals of TC-3 were collected by filtration and recrystallized from ethanol (C_11_H_9_NO_5_; yield, 64.62%; M.p., 91–93°C). IR (KBr, cm^−1^), 1,787 (C=O, lactam) and 1,602 (C=O, amide); ^1^H-NMR (500 MHz; CDCl_3_) δ (ppm), 7.12 (1H, dd, H5), 6.96 (1H, dd, H7), 7.25 (1H, m, H6), 2.71 (3H, s, phenyl-OCOCH_3_) and 2.32 (3H, s, -NCOCH_3_).

#### Determination of compound purity

A Shimadzu HPLC system (Shimadzu, Tokyo, Japan) was utilized for the experiments and consisted of the following components: a 3500 pump, AS 3000 auto sampler, and PDAD 1000 detector. A welchrom-C18, 250×4.6 mm, 5 μm analytical column (Welch Materials, Inc., Potomac, MD, USA) was used. The instrumental settings were: flow rate, 1 ml/min; column oven temperature, 30°C; detector wavelength, 215 nm, 215 nm and 210 nm, respectively; and injection volume, 10 μl. Data acquisition was made using LCsolution version 1.2 (Shimadzu). Peak purities were checked using a photodiode array detector. The mobile phase consisted of methanol and water (50:50, v/v). The mobile phases were filtered through a 0.45-μm nylon filter and degassed. The three compounds were dissolved in the mobile phase.

#### Animals and treatment

Local Kunming mice of both genders, ranging from 18–22 g in weight, were provided by the Experimental Animal Center of Guangxi Medical University (Guangxi, China). This study was conducted in accordance with protocols approved by the Institutional Ethics Committee of Guangxi Medical University.

The mice were divided randomly into various groups containing ten mice in each group: the normal control group, the sodium carboxymethyl cellulose (CMC-Na) group, the CCl_4_ model group, the Bif group and the HBOA, AcO-BOA and TC-3 (high dose, middle dose and low dose) groups. The acute liver injury model was established through the intraperitoneal injection of 0.1 ml/10 g body weight of 0.15% (v/v) CCl_4_ solution in peanut oil to the mice, with the exception of the control and CMC-Na groups. The synthesized compounds were administered orally (at 200, 100 and 50 mg/kg) in 6% CMC solution and the Bif group mice were treated with Bif (150 mg/kg) 8 h later. The mice in the control group received the same volume of normal saline solution, and the CMC-Na and model groups received the same volume of 6% CMC. An additional dose of the treatment was administered after a further 16 h. At 8 h after the last treatment the mice were sacrificed by cervical dislocation. Serum samples were obtained from the eyeballs of the mice and stored at −20°C. The livers were removed from the mice and weighed. Liver tissue (0.2 mg) was accurately weighed, homogenized and centrifuged (1,575 × g for 10 min). The supernatant was separated and stored at −20°C. The remaining liver samples were fixed in 10% formaldehyde for histological analysis.

#### Liver function and biochemical assays

The liver index was calculated for each group using the following formula: Liver index = [mouse liver weight (g)/mouse weight (g)] × 100. The serum levels of ALT and AST were determined using commercially available kits according to the manufacturer's instructions. Levels of LDH in the serum and levels of MDA, SOD, CAT, GSH and Gpx in the liver tissue were measured in accordance with the kit manufacturer's instructions. The inhibition rate of MDA was calculated as follows: (level in the model group - level in the treatment group)/level in the model group × 100. The induction rates of SOD and GSH were calculated as follows: (level in the treatment group - level in the model group)/level in the model group × 100.

#### Histopathology and immunohistochemistry

Liver tissue sections fixed in 10% formaldehyde were embedded in paraffin. Paraffin sections were stained with hematoxylin and eosin (H&E). The TNF-α levels in the liver tissues were measured using an immunohistochemical assay. The degree of liver damage was examined blindly using an Olympus CX41 biological microscope (Tokyo, Japan).

#### Statistical analysis

Statistical analysis was performed using SPSS 13.0 for Windows (SPSS, Inc., Chicago, IL, USA). One way analysis of variance (ANOVA) was used to compare the means among the groups. Data are presented as the means ± SE. P<0.05 was considered to indicate a statistically significant difference.

## Results

### Chemistry

HBOA and its derivatives were synthesized and the purities of the products were determined. The chemical structures of the products are presented in Fig. 1B. The purities of HBOA, AcO-BOA and TC-3 were 99.26, 94.42 and 93.73%, respectively.

### Pharmacology

#### Liver index

The liver index in the model group was significantly higher compared with that of the normal control group. However, the liver indices in the high-dose HBOA and AcO-BOA and mid- and high-dose TC-3 groups were markedly reduced compared with that of the model group (Table I).

#### Effect of HBOA

**Figure 2:**
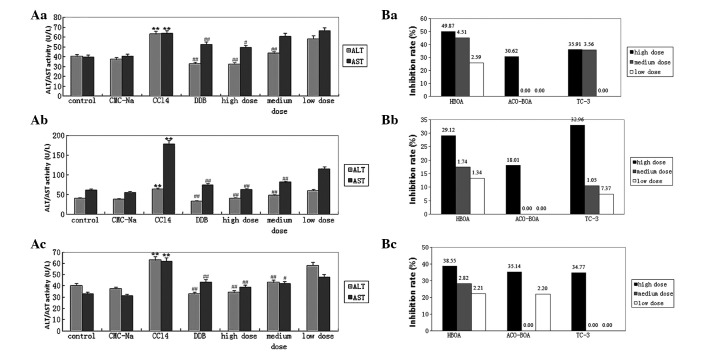
(A) Effects of (a) HBOA, (b) AcO-BOA and (c) TC-3 on serum ALT and AST levels. Data are presented as the means ± SE (n=10). ^**^P<0.01 compared with the normal control group; ^#^P<0.05 and ^##^P<0.01 compared with the CCl_4_ group. (B) Comparison of (a) the MDA inhibition rate and the induction rates of (b) SOD and (c) GSH following treatment with HBOA, AcO-BOA or TC-3 in mice with acute liver injury. Results are presented as: (level in the model group - level in the treatment group)/level in the model group × 100 and (level in the treatment group - level in the model group)/level in the model group × 100, respectively. HBOA, 4-hydroxy-2(3H)-benzoxazolone; AcO-BOA, 4-acetoxy-2(3H)-benzoxazolone; TC-3, 3-acetyl-4-acetoxy-2-benzoxazolone; ALT; alanine aminotransferase; AST, aspartate aminotransferase; MDA, malondialdehyde; SOD, superoxide dismutase; GSH, glutathione.

**Figure 3:**
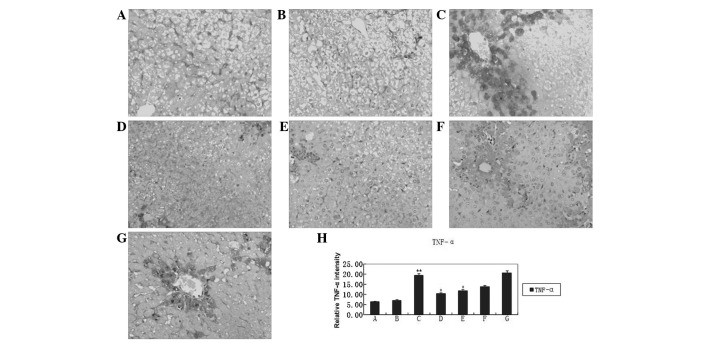
Effects of HBOA on the expression of TNF-α in the liver tissues of mice with acute liver injury (magnification, ×400; Immunohistochemical stain for TNF-α diluted 1:150).(A) NC group; (B) CMC-Na group; (C) CCl_4_ model group; (D) Bif group; (E) high-dose HBOA group; (F) mid-dose HBOA group; (G) low-dose HBOA group. (H) The quantification of TNF-α staining. Data are presented as the means ± SE (n=10). ^**^P<0.01 compared with the NC group; ^#^P<0.05 compared with the CCl_4_ group. HBOA, 4-hydroxy-2(3H)-benzoxazolone; TNF-α, tumor necrosis factor-α; NC, normal control; CMC-Na, sodium carboxymethyl cellulose; Bif, bifendate.

The levels of serological indicators (AST, ALT, MDA) were significantly higher in the model group compared with the normal control group. Compared with the values in the model group, high-dose HBOA induced significant reductions in the activities of ALT and AST and increases in the activities of SOD, Gpx and CAT, and mid- and high-dose HBOA induced significant reductions in the levels of MDA and LDH and increases in the activity of GSH (Fig. 2Aa; Table II). The expression of TNF-α was significantly reduced by high-dose HBOA (Fig. 3).

#### Effect of AcO-BOA

Acute liver injury induced by CCl_4_ provoked significant reductions in the activities of liver SOD and GSH, and increased the liver MDA and serum ALT and AST levels. The results demonstrated that the activities of liver SOD and GSH were increased by treatment with high-dose AcO-BOA, whereas the liver MDA and serum ALT and AST levels were markedly reduced (Fig. 2Ab; Table III).

#### Effect of TC-3

CCl_4_ intoxication reduced the activities of SOD and GSH and raised the levels of ALT, AST and MDA. Conversely, in mice treated with TC-3, the levels of AST and MDA were reduced and SOD and GSH levels were elevated (Fig. 2Ac; Table IV).

#### Inhibition rate of MDA and induction rate of SOD and GSH

A high-dose of each of the three compounds demonstrated the highest MDA inhibition rates and the highest SOD and GSH induction rates (Fig. 2B).

#### Histopathological observation

**Figure 4:**
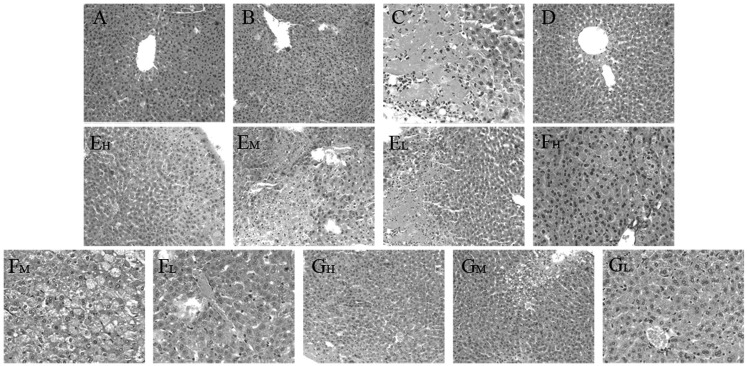
Effects of HBOA and its derivatives on histopathological changes induced by CCl_4_ in the mouse liver. Liver sections were H&E stained (magnification, ×100): (A) normal control group; (B) CMC-Na group; (C) CCl_4_ model group; (D) CCl_4_ + Bif 150 mg/kg group; (E_H_) CCl_4_ + HBOA 200 mg/kg group; (E_M_) CCl_4_ + HBOA 100 mg/kg group; (E_L_) CCl_4_ + HBOA 50 mg/kg group; (F_H_) CCl_4_ + AcO-BOA 200 mg/kg group; (F_M_) CCl_4_ + AcO-BOA 100 mg/kg group; (F_L_) CCl_4_ + AcO-BOA 50 mg/kg group; (G_H_) CCl_4_ + TC-3 200 mg/kg group; (G_M_) CCl_4_ + TC-3 100 mg/kg group; (G_L_) CCl_4_ + TC-3 50 mg/kg group. HBOA, 4-hydroxy-2(3H)-benzoxazolone; H&E, hematoxylin and eosin; AcO-BOA, 4-acetoxy-2(3H)-benzoxazolone; TC-3, 3-acetyl-4-acetoxy-2-benzoxazolone; CMC-Na, sodium carboxymethyl cellulose; Bif, bifendate.

The degree of liver injury observed in mice was evaluated by H&E staining, which demonstrated that the livers from the normal control and CMC-Na control mice exhibited a normal liver lobular architecture with a central vein and radiating hepatic cords (Fig. 4A and B). In contrast to normal mouse liver morphology, CCl_4_-induced injury was evidenced by disruption of the liver lobular architecture, liver cell edema, fatty degeneration of the liver, inflammation and granuloma (Fig. 4C). These alterations were reduced in the liver sections of mice that received the three compounds in high and medium doses (Fig. 4E–G). Concurrent administration of bifendate attenuated the CCl_4_-induced changes in the liver architecture (Fig. 4D).

## Discussion

Regarding the chemical structure of HBOA, it contains hydroxyl and imine bases that may act as either hydrogen bond donors or acceptors and play a role in the action of the drug and in the process of receptor binding. Calış *et al* observed that the substitution of the benzene ring of benzoxazolinone derivatives by an acyl moiety resulted in enhanced anti-inflammatory and analgesic activities (20). Therefore, we hypothesize that 3-substituted or 3,4-disubstituted derivatives of HBOA may possess a greater protective effect on the liver than HBOA.

Products were carbonized by concentrated sulfuric acid. In order to avoid carbonization and maintain the concentration of sulfuric acid, we added distilled water or filtrate when distilling. We obtained 2-nitroresorcinol by drip type water vapor distillation in order to avoid carbonising the product. HBOA was obtained from 2-nitroresorcinol by a ‘one-pot’ reduction reaction and subsequent cyclization with urea. In this reaction, hexahydrated ferric chloride-activated carbon was used as a catalytic agent and hydrazine hydrate was used as a reducing agent. We have previously compared the applicability of reduction with hydrazine hydrate with that of stannous chloride dissolved in hydrochloric acid; reduction by hydrazine hydrate was observed to be simpler and more convenient (21). However, hydrazine hydrate must be evaporated to dryness to prevent it from reacting with urea. AcO-BOA and TC-3 were subsequently obtained by the reaction of HBOA with acetyl chloride, an acylating agent, in a basic triethylamine solution.

HPLC was used to determine the purity of the compounds. The method provides excellent separation of target compounds from unknown impurities with a resolution of >1.5 and a tailing factor range of 0.95–1.5. We observed that AcO-BOA and TC-3 are readily hydrolyzed in aqueous solution. Therefore, we employed a solution of CMC as required in order to avoid hydrolysis.

CCl_4_-induced liver injury is dependent upon reductive dehalogenation catalyzed by cytochrome p450 in the endoplasmic reticulum of hepatic cells leading to the generation of an unstable complex trichloromethyl radical (^•^CCl_3_). The superoxide anion O_2_^−^, H_2_O_2_ and the hydroxyl radical (^•^OH) are reactive oxygen species (ROS) produced in mitochondria (4). Lipid peroxidation is an important consequence of the metabolism of CCl_4_(22). These oxygen radicals contribute to the process of lipid peroxidation. However, cells have various mechanisms for protecting themselves from the toxic effects of ROS, including free radical scavengers and chain reaction terminators such as SOD, CAT, GSH and Gpx systems (23). Liver injury via the ROS pathway causes increases in the levels of ALT, AST, MDA and LDH, the syntheses of which are increased due to the extensive damage of the liver cells (4,24).

The current study demonstrates that HBOA and its derivatives exhibit protective effects against acute liver injury, confirmed by the serum ALT and AST levels and H&E staining. Our investigation revealed that HBOA and its derivatives exhibit potent hepatoprotective effects against CCl_4_-induced liver damage in mice. This may be the result of increasing the activity of the antioxidant-defense system and the inhibition of lipid peroxidation.

Many hepatoprotective drugs have been of interest due to their antioxidant activity (25). In the current study, the induction of acute liver injury by CCl_4_ increased the levels of MDA in liver tissue. MDA is a product of lipid peroxidation; elevated levels of MDA may reflect the degree of lipid peroxidation injury in liver cells (2). High doses of HBOA and its derivatives are able to reduce the levels of hepatic MDA. Compared with the CCl_4_ model group, treatment with HBOA increased the activity or level of SOD, CAT, Gpx and GSH, which scavenge free radicals and simultaneously reduce lipid peroxidation, thus alleviating the oxidative damage caused by CCl_4_. The results suggest that HBOA protects against the damage caused by the oxidation of hepatic cellular membranes by free radical scavenging. Treatment of the mice with the two derivatives of HBOA attenuated the hepatic SOD and GSH depletion induced by the intraperitoneal administration of CCl_4_. Therefore, we hypothesize that the protective actions of the two derivatives are related to their ability to scavenge free radicals.

TNF-α activates various intracellular pathways which regulate inflammation, cell death and proliferation. In the liver, TNF-α not only mediates hepatotoxicity but also contributes to the restoration of functional liver mass by promoting hepatocyte proliferation and liver regeneration (26). TNF-α expression was observed to be lower in the group treated with HBOA than in the model group, which demonstrates that the hepatoprotective effect of HBOA involved an anti-inflammatory mechanism and inhibiting the activity of secreted TNF-α on Kupffer cells (KCs).

In conclusion, these preliminary studies indicate that HBOA and its two acyl derivatives elicit a protective effect on CCl_4_-induced liver injury *in vivo*. This study supports the traditional use of *Acanthus ilicifolius* as an herbal remedy for various liver diseases. However, further studies are required in order to evaluate the mechanism of action these compounds.

## Figures and Tables

**Table I:** Effect of HBOA, AcO-BOA and TC-3 on the liver index in mice (%).

Group	HBOA	AcO-BOA	TC-3
Control	4.56±0.51	4.93±0.41	5.59±0.34
CMC-Na	4.60±0.36	5.17±0.59	5.35±0.49
CCl_4_	5.28±0.45^a^	5.66±0.75^b^	6.06±0.46^a^
Bif	4.61±0.35^c^	4.83±0.30^c^	5.29±0.18^d^
High-dose	4.73±0.44^d^	4.88±0.49^d^	5.43±0.38^d^
Med-dose	5.38±0.79	5.23±0.59	5.44±0.20^d^
Low-dose	5.77±0.86	5.84±0.98	5.52±0.47^c^

Data are presented as the means ± SE. n=10 per group.

a P<0.01 and

b P<0.05 compared with the normal control group;

c P<0.01 and

d P<0.05 compared with the CCl_4_ group.

HBOA, 4-hydroxy-2(3H)-benzoxazolone; AcO-BOA, 4-acetoxy-2(3H)-benzoxazolone; TC-3, 3-acetyl-4-acetoxy-2-benzoxazolone; CMC-Na, sodium carboxymethyl cellulose; Bif, bifendate.

**Table II:** Effect of HBOA on SOD, MDA, GSH, Gpx, CAT and LDH levels (n=10).

Group	SOD (U/mgprot)	MDA (nmol/mgprot)	GSH (nmol/mgprot)	Gpx (U/mgprot)	CAT (U/mgprot)	LDH (U/l)
Control	200.84±41.95	2.20±0.60	2.20±0.83	368.74±101.27	12.74±3.29	5423.47±1054.40
CMC-Na	215.18±43.38	2.36±0.93	2.95±1.41	374.33±103.56	11.30±5.53	5942.01±1074.70
CCl_4_	139.49±30.52^a^	3.79±1.74^a^	1.02±0.36^a^	269.06±61.43^a^	8.18±2.82^a^	7907.96±2420.00^a^
Bif	191.61±36.57^c^	1.88±0.78^c^	1.63±0.89^b^	344.46±72.22^b^	11.41±1.81^c^	5967.66±1305.49^b^
High-dose HBOA	196.81±27.16^c^	1.90±0.91^c^	1.66±1.00^b^	341.57±76.65^b^	12.92±5.41^b^	5288.04±1151.85^c^
Med-dose HBOA	168.85±42.47	2.08±0.49^c^	1.42±0.53^b^	263.04±81.16	11.38±6.18	5764.20±865.38^b^
Low-dose HBOA	160.99±44.77	2.81±1.89	1.31±0.91	278.41±68.83	7.25±3.25	7172.09±370.51

Data are presented as the means ± SE.

a P<0.01 compared with the normal control group;

b P<0.05 and

c P<0.01 compared with the CCl_4_ group.

HBOA, 4-hydroxy-2(3H)-benzoxazolone; SOD, superoxide dismutase; MDA, malondialdehyde; GSH, glutathione; Gpx, glutathione peroxidase; CAT, catalase; LDH, lactic dehydrogenase, CMC-Na, sodium carboxymethyl cellulose; Bif, bifendate.

**Table III:** Effect of high-, mid- and low-dose AcO-BOA on SOD, MDA and GSH levels (n=10).

Group	SOD (U/mgprot)	MDA (nmol/mgprot)	GSH (nmol/mgprot)
Control	291.51±92.07	1.59±0.48	4.42±2.32
CMC-Na	305.37±84.18	1.89±0.59	3.68±1.59
CCl_4_	203.72±49.49^a^	2.58±0.85^b^	2.16±0.78^b^
Bif	269.34±82.90^c^	1.32±0.47^d^	3.27±0.86^d^
High-dose	288.98±102.00^c^	1.79±0.70^c^	3.33±0.95^d^
Mid-dose	217.58±74.44	2.65±0.67	1.11±0.56
Low-dose	227.66±56.68	2.80±1.04	2.77±0.15

Data are presented as the means ± SE.

a P<0.05 and

b P<0.01 compared with the normal control group;

c P<0.05 and

d P<0.01 compared with the CCl_4_ group.

AcO-BOA, 4-acetoxy-2(3H)-benzoxazolone; SOD, superoxide dismutase; MDA, malondialdehyde; GSH, glutathione; CMC-Na, sodium carboxymethyl cellulose; Bif, bifendate.

**Table IV:** Effect of high-, mid- and low-dose TC-3 on SOD, MDA and GSH levels (n=10).

Group	SOD (U/mgprot)	MDA (nmol/mgprot)	GSH (nmol/mgprot)
Control	246.81±86.41	2.81±0.62	4.57±2.30
CMC-Na	262.09±33.99	2.94±0.23	4.96±1.07
CCl_4_	193.37±22.36^a^	3.62±0.92^a^	2.57±0.84^a^
Bif	290.39±63.25^b^	2.74±0.90^b^	4.81±1.43^c^
High-dose	288.45±42.52^c^	2.32±0.65^c^	3.94±1.14^c^
Mid-dose	216.15±20.75^c^	2.33±0.45^c^	2.32±1.05^c^
Low-dose	208.76±54.45	4.30±1.35	2.19±1.15^c^

Data are presented as the means ± SE.

a P<0.05 compared with the normal control group;

b P<0.05 and

c P<0.01 compared with the CCl_4_ group.

TC-3, 3-acetyl-4-acetoxy-2-benzoxazolone; SOD, superoxide dismutase; MDA, malondialdehyde; GSH, glutathione; CMC-Na, sodium carboxymethyl cellulose; Bif, bifendate.

## Abbreviations

CCl_4_: carbon tetrachloride
ALT: alanine aminotransferase
AST: aspartate aminotransferase
LDH: lactic dehydrogenase
MDA: malondialdehyde
SOD: superoxide dismutase
CAT: catalase
GSH: glutathione
Gpx: glutathione peroxidase
